# A New UPLC-qTOF Approach for Elucidating Furan and 2-Methylfuran Metabolites in Human Urine Samples after Coffee Consumption

**DOI:** 10.3390/molecules25215104

**Published:** 2020-11-03

**Authors:** Simone Stegmüller, Nadine Beißmann, Jonathan Isaak Kremer, Denise Mehl, Christian Baumann, Elke Richling

**Affiliations:** 1 Technische Universität Kaiserslautern, Department of Chemistry, Division of Food Chemistry and Toxicology, Erwin-Schrödinger-Str. 52, 67663 Kaiserslautern, Germany; stegmueller@chemie.uni-kl.de (S.S.); beissmann@chemie.uni-kl.de (N.B.); kremer@chemie.uni-kl.de (J.I.K.); 2 AB SCIEX Germany GmbH, 64293 Darmstadt, Germany; denise.mehl@sciex.com (D.M.); Christian.baumann@sciex.com (C.B.)

**Keywords:** furan, 2-methylfuran, UPLC-qToF, untargeted analysis, urinary metabolites

## Abstract

We have investigated urine samples after coffee consumption using targeted and untargeted approaches to identify furan and 2-methylfuran metabolites in urine samples by UPLC-qToF. The aim was to establish a fast, robust, and time-saving method involving ultra-performance liquid chromatography-quantitative time-of-flight tandem mass spectrometry (UPLC-qToF-MS/MS). The developed method detected previously reported metabolites, such as Lys-BDA, and others that had not been previously identified, or only detected in animal or in vitro studies. The developed UPLC-qToF method detected previously reported metabolites, such as lysine-*cis*-2-butene-1,4-dial (Lys-BDA) adducts, and others that had not been previously identified, or only detected in animal and in vitro studies. In sum, the UPLC-qToF approach provides additional information that may be valuable in future human or animal intervention studies.

## 1. Introduction

Furan and 2-methylfuran (2-MF) are colorless liquids that are very slightly soluble in water. Furan has been classified by the International Agency for Research on Cancer (IARC) as a possible carcinogen (class 2B) [1,2].

Furan is mainly found in coffee and jarred food. Therefore, in Europe adults and infants reportedly have the highest furan intakes, and 95% of the adults’ daily intake is reportedly through coffee, as the mean level of furan in whole roasted coffee beans is 4.58 mg/kg [3]. The furan content of coffee beverages depends on various factors, such as roasting temperature and duration, preparation method, and use of instant or ground coffee. Coffee beverages prepared by an espresso machine have higher furan levels than coffee prepared, from the same ground coffee, by a drip coffee maker (open system) [4,5]. The 2-MF content has been found to be ca. six-fold higher than the furan content and is the main furan derivative identified in coffee [6].

Furan and 2-MF are formed in food via various pathways and precursors, such as ascorbic acid, amino acids, carbohydrates, unsaturated fatty acids, and carotenoids. Furan formation also occurs during the thermal decomposition of serine and cysteine [7,8,9]. Furthermore, 2-MF can be formed from amino acid and α,β-unsaturated aldehydes [9].

There is no experimental evidence of furan genotoxicity in peripheral blood or bone marrow cells but chromosomal aberrations linked to its exposure have been detected in rat splenocytes, indicating that it may damage DNA in the liver within 28 days [10]. Moreover, in a two-year study administration by gavage reportedly increased incidences of mononuclear cell leukemia, cholangiocarcinoma, and hepatocellular neoplasms in livers of male and female rats [11]. However, hepatotoxicity and carcinogenicity depend on doses and time [12], and for hepatocarcinogenicity furan must be activated to *cis*-2-butene-1,4-dial (BDA) by cytochrome P450 2E1 [13].

Bioactivation also seems to be necessary for 2-MF activity [14]. In hepatic and pulmonary microsomal systems acetylacrolein (AcA; (2Z)-oxopent-2-enal) has been identified as a possible reactive intermediate of 2-MF [15]. Apoptotic hepatocytes, abnormally pigmented Kupffer cells, and inflammatory infiltration have been observed in rats after 2-MF intake [16], and a No Observed Adverse Effect Level (NOAEL) of 0.4 mg/kg bodyweight (BW) per day has been calculated for it [6].

Due to the high reactivity of the main metabolites, BDA and AcA, of furan and 2-MF, they can react in the human body with diverse biomolecules, such as amino acids, glutathione, and nucleosides. Thus, the aims of this study were to identify adducts of BDA and AcA in the urine of participants who had previously consumed a coffee brew and establish excretion kinetics for identified metabolites. Since the examination and evaluation of the collected urine samples with a targeted approach using conventional high-performance liquid chromatography-tandem mass spectrometry (HPLC-MS/MS) analysis would be very time-consuming and costly, a new approach using ultra-performance LC-quantitative time-of-flight MS/MS was applied. This approach requires less laborious sample preparation, and provides exact masses of analytes, with the associated possibility of database searches, recording all MS/MS spectra over the set mass range, as well as a significant reduction in measuring time. The urine samples tested had already been examined in a previous study with a targeted approach [17,18]. The results obtained in the study presented here are intended to complement these data by identifying previously unknown metabolites and show whether the new approach can provide at least as good results as the targeted approach.

## 2. Results and Discussion

The main objective of the study was to determine the effectiveness of the untargeted UPLC-qToF-MS/MS approach for identifying known and unknown metabolites in human urine without complex sample preparation and the possibility of using it to complement or even replace targeted UPLC-MS/MS analyses. A major advantage of the method is that a large amount of data is collected for each sample and can be retrospectively used to address various questions.

The specific aims of this study were to identify furan and 2-MF in metabolites in samples of urine collected from participants 36 h after they had consumed a bolus of coffee brew. Details of the study design have been previously published [17]. Due to limitations in time to use the qToF apparatus, only urine samples from five of the 10 participants were used in the UPLC-qToF-MS/MS measurements. The results were compared with results from previous targeted investigations. An overview of the identified metabolites of furan and 2-methylfuran are given in Table 1 and Table 2. 

### 2.1. Identification of Furan-Derived Metabolites

**Figure 1:**
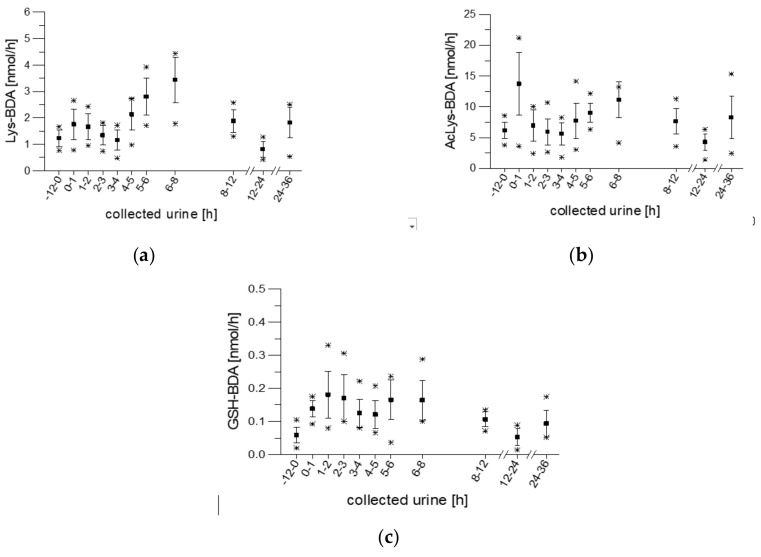
Excretion kinetics of Lys-BDA (**a**), AcLys-BDA (**b**), and GSH-BDA (**c**): HPLC-ESI-MS/MS measurements of contents in samples of urine from participants 1 to 10 at indicated times after coffee consumption (h). Data are previously published means with standard deviations and minimum/maximum values [20].

In previous studies by our group, the metabolites lysine-BDA (Lys-BDA), acetyl lysine-BDA (AcLys-BDA), and glutathione-BDA (GSH-BDA) were identified and quantified in samples of urine collected from the participants at various times after coffee intake [20]. The resulting kinetics are shown in Figure 1.

**Figure 2:**
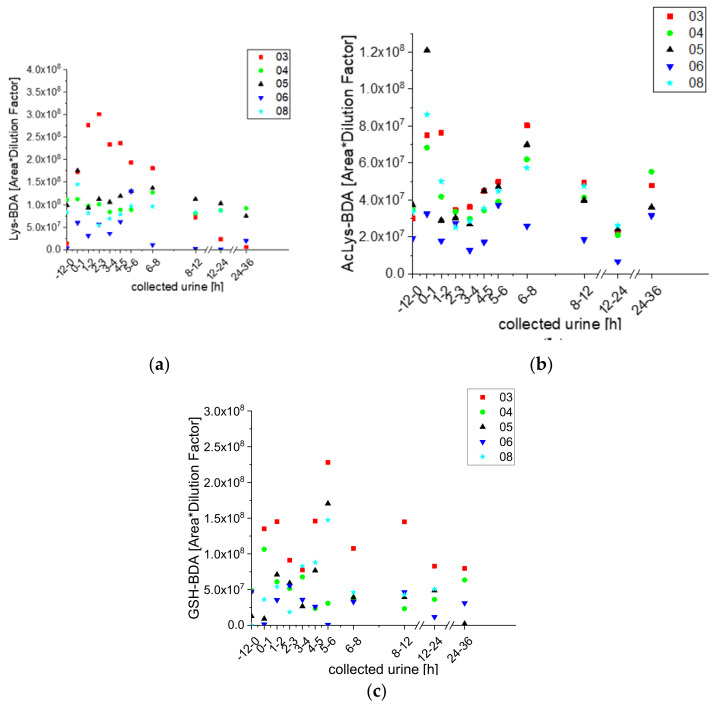
Excretion kinetics of Lys-BDA (**a**), AcLys-BDA (**b**), and GSH-BDA (**c**): UPLC-qToF-MS/MS measurements (as detailed in Materials and Methods) of contents in samples of urine from participants three, four, five, six, and eight at indicated times after coffee consumption (h). Excretion kinetics of Lys-BDA, AcLys-BDA, and GSH-BDA measured by UPLC-qToF (details see Materials and Methods section) over 36 h after coffee consumption. Shown are the individual kinetics of the participants three, four, five, six, and eight. The measurements were performed as triplicates and the data points are shown as mean values with standard deviation.

With the UPLC-qToF-MS/MS analysis, we detected Lys-BDA, NAcLys-BDA, and BDA-GSH in at least one urine sample from each participant at each selected time point, and observed similar changes with time in their concentrations (see Figure 2).

**Figure 3:**
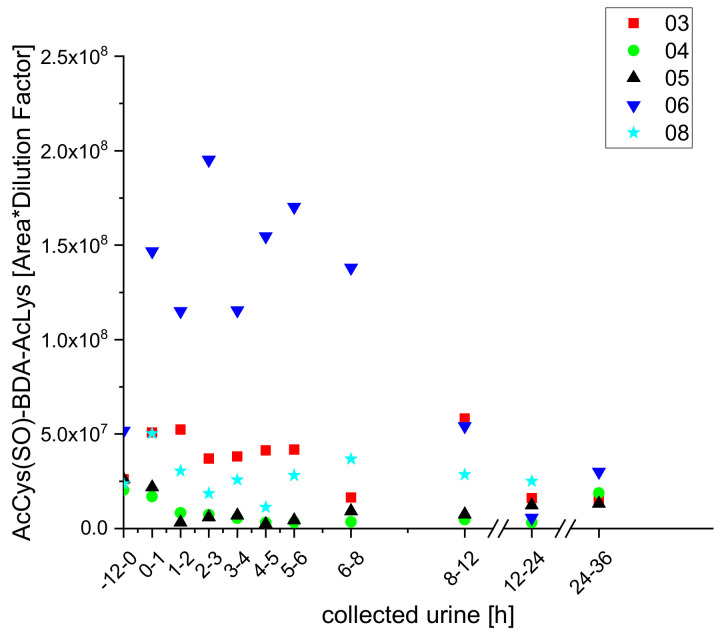
Excretion kinetics of AcCys-(SO)-BDA-AcLys (Rt = 4.46 min): UPLC-qToF-MS/MS measurements (as detailed in Section 3) of contents in samples of urine from participants three, four, five, six, and eight at indicated times after coffee consumption (h).

**Figure 4:**
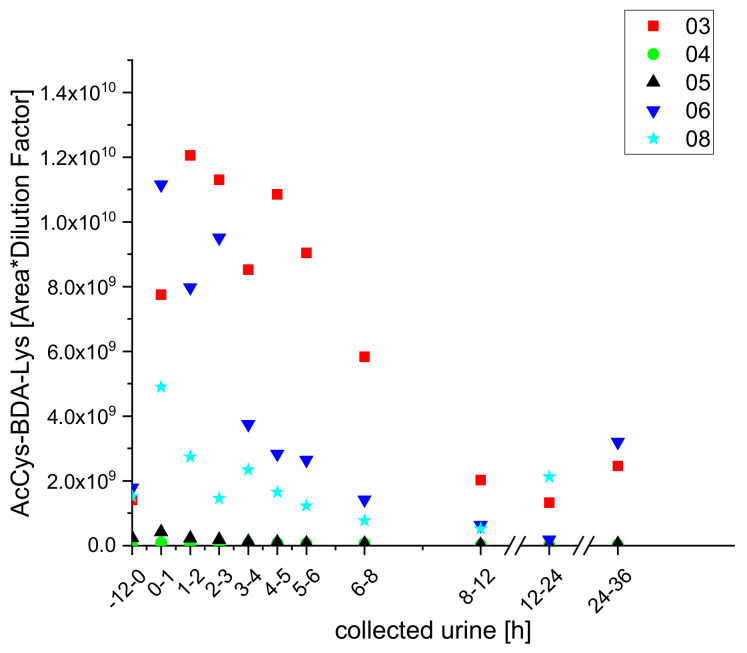
Excretion kinetics of AcCys-BDA-AcLys: UPLC-qToF-MS/MS measurements of contents in samples of urine from participants three, four, five, six, and eight at indicated times after coffee consumption (h).

Using UPLC-qToF-MS/MS, we were able to determine exact masses and obtained MS^2^-spectra for all three metabolites, which enabled us to confirm their structures. Furan-derived metabolites that have been detected in urine samples from rats after furan exposure were also detected in the urine samples of the participants using this approach. These included acetylcysteine butendial lysine (AcCys-BDA-Lys), acetylcysteine-SO-butendial acetyllysine (AcCys-(SO)-BDA-AcLys), methanethiol butendial glutamic acid (methanethiol-BDA-Glu), glutathione butendial lysine (GSH-BDA-Lys), and glutathione butendial glutamine (GSH-BDA-Gln). The metabolites AcCys-BDA-Lys and AcCys-(SO)-BDA-AcLys have already been detected in urine from smokers [23] but could not be quantified in the cited study as the signals were below the respective limits of quantification (LOQs). In the study presented here, we identified both metabolites by exact masses and MS^2^ spectra, and obtained semi-quantitative kinetics, which correlated with the coffee intake, for the two metabolites in the urine samples of five participants (numbers three to six and eight; as shown in Figure 3 and Figure 4).

Both AcCys-(SO)-BDA-AcLys and AcCys-BDA-AcLys were identified in samples from each of the five subjects included in the study presented here but the excretion kinetics differed, so excretion kinetic data for each individual are displayed rather than mean values for all five subjects.

The metabolite methanethiol-BDA-Glu has also been previously detected in rat urine [19] and we detected it in the urine of some of our participants but at lower levels than the other identified metabolites (too low to establish excretion kinetics).

**Figure 5:**
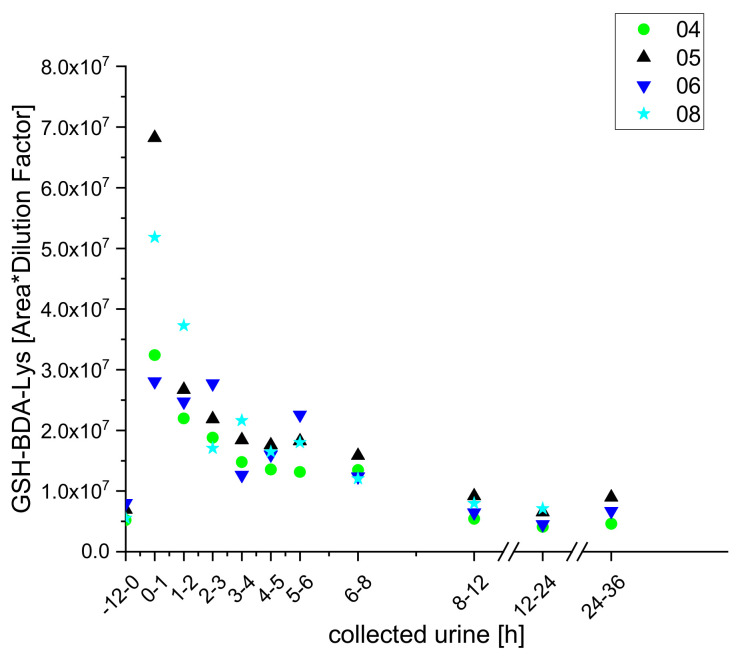
Excretion kinetics of GSH-BDA-Lys: UPLC-qToF-MS/MS measurements of contents in samples of urine from participants four, five, six, and eight at indicated times after coffee consumption (h).

**Figure 6:**
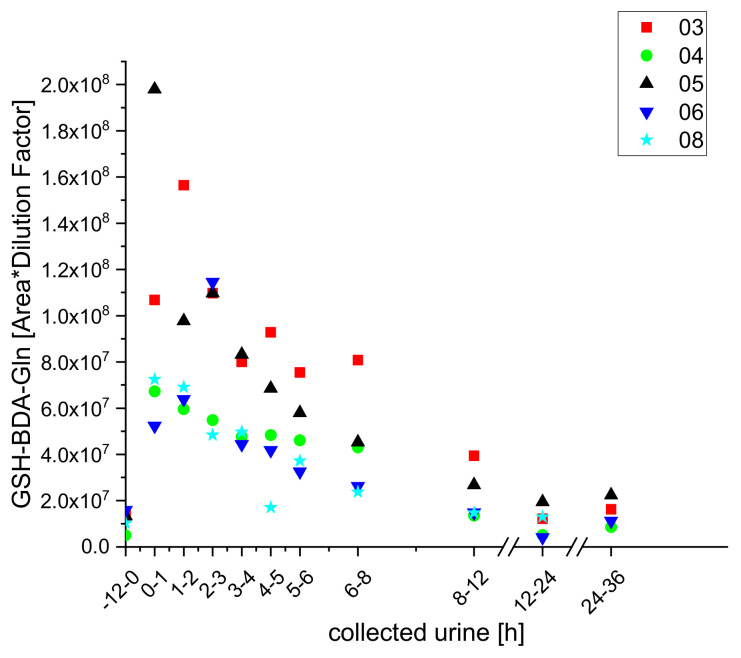
Excretion kinetics of GSH-BDA-Gln: UPLC-qToF-MS/MS measurements of contents in samples of urine from participants three, four, five, six, and eight at indicated times after coffee consumption (h). Excretion kinetics of the GSH-conjugate GSH-BDS-Lys from all five subjects were similar: in all cases, levels in the urine samples increased to a maximum within the first hour then continuously decreased until they reached the initial baseline level after 24 h. In contrast, there were clear between-individual differences in excretion kinetics of GSH-BDA-Gln. Levels of this metabolite peaked in urine from subjects four and eight within the first hour, but only after 3 h in urine from subjects five and six. Moreover, it was not detected at all in urine from participant three.

However, we did detect two GSH-conjugates (GSH-BDA-Lys and GSH-BDA-Gln) that have been previously detected in hepatocytes and rat urine [19,25] and obtained semi-quantitative kinetics for them (Figure 5 and Figure 6).

**Figure 7:**
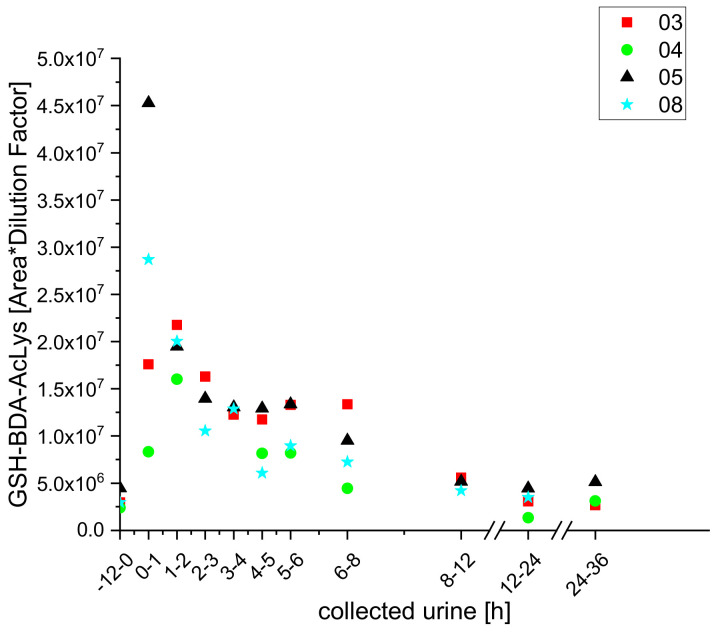
Excretion kinetics of GSH-BDA-AcLys: UPLC-qToF-MS/MS measurements of contents in samples of urine from participants three, four, five, and eight at indicated times after coffee consumption (h).

**Figure 8:**
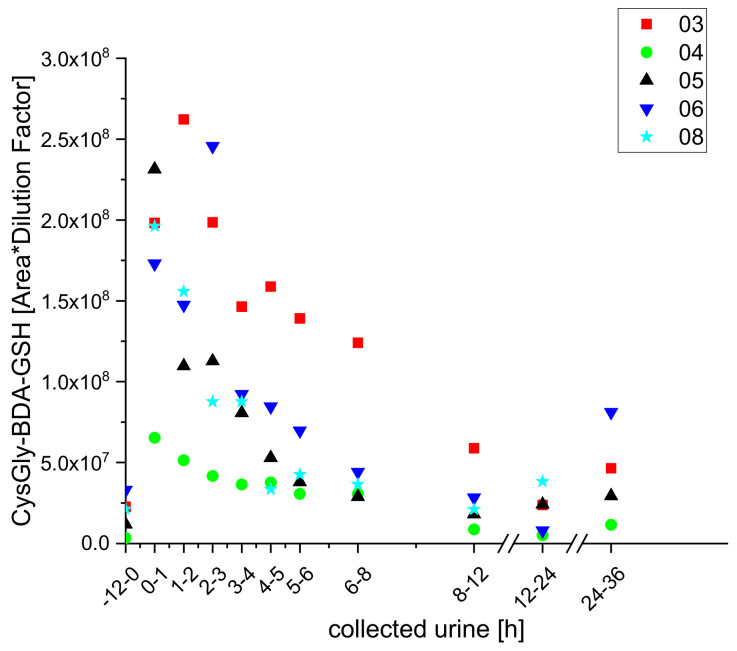
Excretion kinetics of CysGly-BDA-GSH: UPLC-qToF-MS/MS measurements of contents in samples of urine from participants three, four, five, six, and eight at indicated times after coffee consumption (h).

Using UPLC-qToF-MS/MS, we also identified furan metabolites in the urine of the participants that had previously only been detected in vitro or in the bile of rats [19]. These included the GSH adducts glutathione butendial acetyl lysine (GSH-BDA-AcLys), glutathione butendial glutamic acid (GSH-BDA-Glu) cysteine butendial glutathione (Cys-BDA-GSH), and cysteine glycine butendial glutathione (Cys-Gly-BDA-GSH). All of these were identified, by exact masses and MS^2^ spectra, for the first time in urine samples of human subjects, and coffee consumption-dependent kinetics were observed for two of them (GSH-BDA-AcLys and CysGly-BDA-GSH), as shown in Figure 7 and Figure 8.

For GSH-BDA-Ac-Lys, we obtained similar initial values (in urine samples collected during the 12 h before coffee consumption) for all subjects. Moreover, its levels peaked in samples from all participants within the two hours following coffee consumption and declined to the baseline level within 24 h. 

However, there was large variation in the triplicate determinations for samples from participant 6, due to low concentrations of the metabolite, and high uncertainties, so we excluded these data from further analysis.’ 

**Figure 9:**
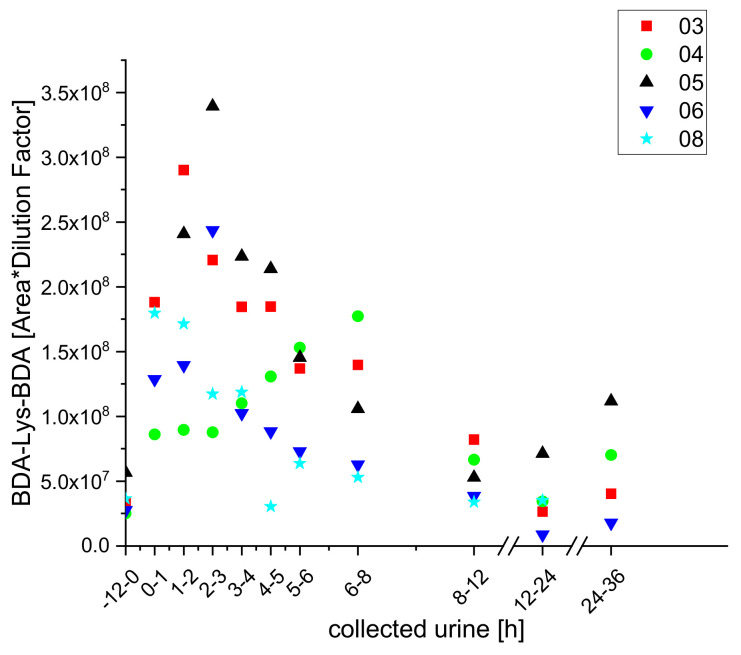
Excretion kinetics of BDA-Lys-BDA: UPLC-qToF-MS/MS measurements of contents in samples of urine from participants three, four, five, six, and eight at indicated times after coffee consumption (h).

Furthermore, we identified metabolites in our urine samples, which have not been previously described in the literature at all: a biadduct of BDA with lysine (BDA-Lys-BDA) and a BDA cysteine adduct (Cys-BDA). Levels of the BDA biadduct were clearly related to the coffee intake (Figure 9).

**Figure 10:**
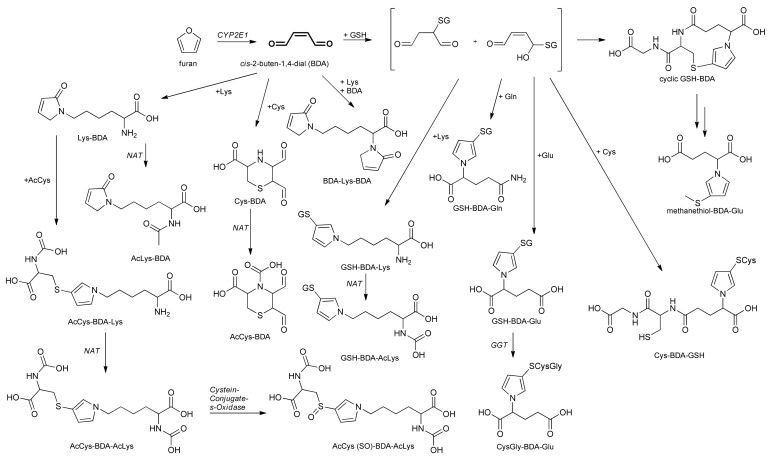
Proposed metabolic pathways for furan based on metabolites identified by UPLC-qToF-MS/MS in samples of urine from human participants after consumption of a bolus of coffee brew and previously identified pathways [19].

Based on our findings regarding metabolites detected by the UPLC-qToF-MS/MS approach in human urine samples, we propose the metabolic pathways for furan taken in by the ingestion of a coffee brew shown in Figure 10.

### 2.2. Identification of 2-Methylfuran Derived Metabolites

**Figure 11:**
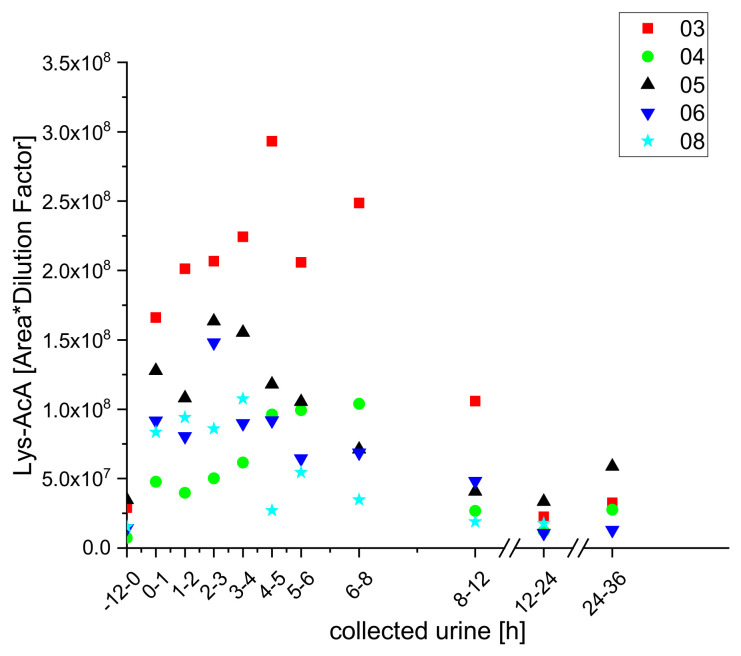
Excretion kinetics of Lys-AcA: UPLC-qToF-MS/MS measurements of contents in samples of urine from participants three, four, five, six, and eight at indicated times after coffee consumption (h).

**Figure 12:**
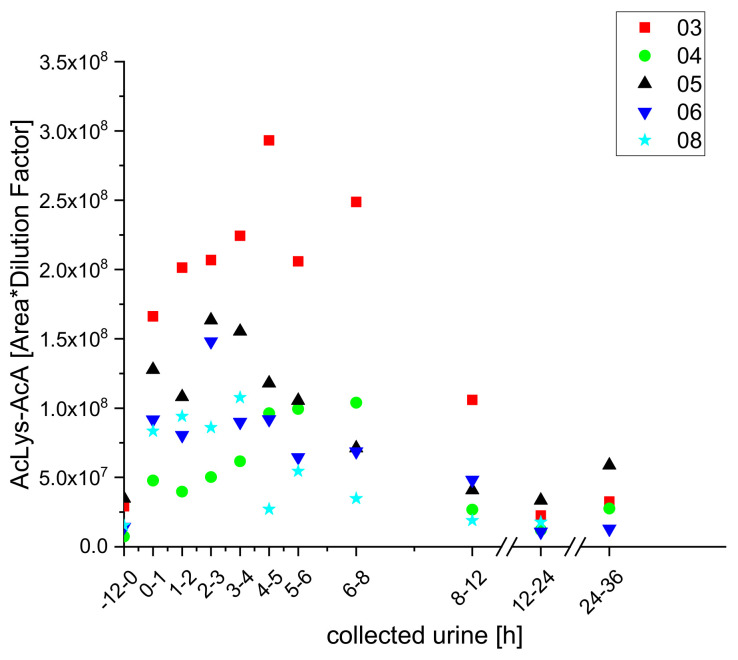
Excretion kinetics of AcLys-AcA: UPLC-qToF-MS/MS measurements of contents in samples of urine from participants three, four, five, six, and eight at indicated times after coffee consumption (h).

In analogy to the furan metabolites, we detected two 2-methylfuran conjugates with lysine (Lys-AcA) and acetyl lysine (AcLys-AcA) in samples of urine from the test subjects after coffee brew intake (Figure 11 and Figure 12).

**Figure 13:**
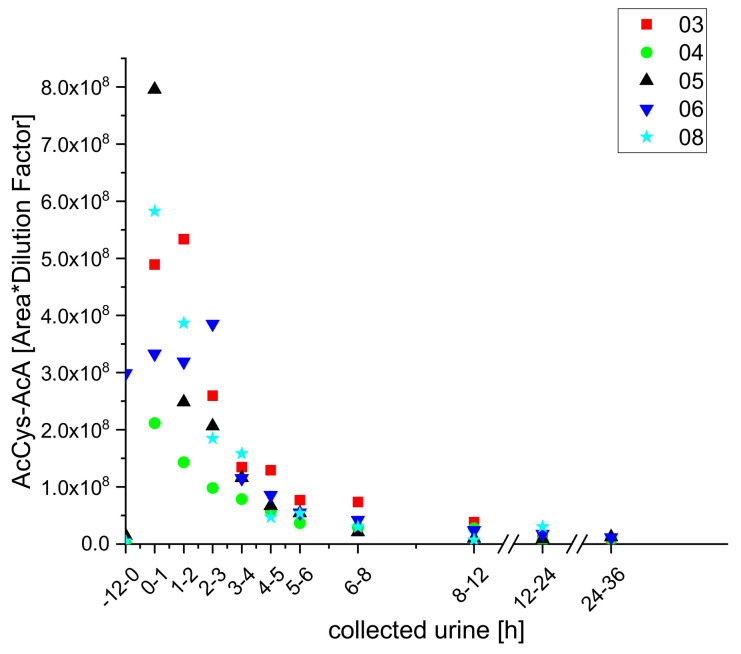
Excretion kinetics of AcCys-AcA: UPLC-qToF-MS/MS measurements of contents in samples of urine from participants three, four, five, six, and eight at indicated times after coffee consumption (h).

To our surprise, we also identified a conjugate of AcA and acetylcysteine, acetylcysteine acetylacrolein (AcCys-AcA), which has only been previously postulated as a theoretical metabolite, and observed kinetic changes in its levels that were strongly dependent on coffee consumption (Figure 13).

## 3. Materials and Methods

### 3.1. Chemicals and Reagents

Acetonitrile and formic acid (both LC-MS grade) were purchased from Honeywell (Charlotte, North Carolina, USA), and Biosolve (Valkenswaard, The Netherlands), respectively. Double distilled water produced with a BÜCHI (Essen, Germany) unit was used for the solvents and to dilute the urine samples.

### 3.2. Human Urine Samples

The analyzed urine specimens were obtained in a previously described human intervention study [17]. The study, which was approved by the ethical committee of the Rhineland-Palatinate medical commission (Approval No. 837.427.15(10195)) was performed in accordance with ethical standards of the Declaration of Helsinki (1964) [27]. For the exploratory investigation presented here, samples from five of the 10 subjects were examined. Portions of urine samples collected at time points indicated in the figures were membrane-filtered, transferred to autosampler vials, and subjected to UPLC-qToF-MS/MS analyses.

### 3.3. Liquid Chromatography-High Resolution Mass Spectrometry (LC-HRMS)

The urine samples were analyzed using a ExionLC^TM^ AD System (Darmstadt, Germany) coupled with a TripleTOF^®^ 6600 system from Sciex. The UPLC conditions were chosen to detect the broadest possible range of metabolites. They were separated using a Synergi 4 u polar RP column (100 × 2.00 mm, Phenomenex, Torrance, CA, USA) with a column oven temperature of 20 °C and a mobile phase consisting of a mixture of 0.1% aqueous formic acid (A) and acetonitrile (B): 1% B for 0.1 min, linear increases to 30% B from 0.1 to 5.8 min, then to 80% B within 0.1 min, followed by a hold of 80% B for 2 min and re-equilibration to 1% B for a further 2 min (with a constant flow rate of 300 µl min^-1^). Eluting analytes were quantified by the MS system operating in both positive and negative ionization modes. The conditions for positive mode were: curtain gas (CUR) 30 psi, ion source gas (GS) 1 and 2 pressure 60 and 70 psi, temperature 500 °C, ion spray voltage floating (ISVF) 5000 V. Conditions for negative mode were: CUR 40 psi, GS 1 and 2 pressure 60 and 70 psi, temperature 650 °C, ISVF 4000 V. First acquisitions of mass spectra were performed using positive and negative ionization modes with information-dependent acquisition (IDA) of product ions. In full-scan mode, the accumulation time was 100 ms for a mass range from 60 to 800 Da. Each IDA experiment had an accumulation time of 60 ms with the collision energy set to −35 or 35 eV with a spread of 15 eV in high-resolution mode. The IDA criteria were as follows: the 15 most intense ions (number of IDA experiments) with an intensity threshold of 200 cps and exclusion time of 5 s after one occurrence. In second acquisitions, spectra were acquired data-independently by the “sequential window acquisition of all theoretical fragment-ion spectra” (SWATH^®^) method with positive and negative ionization. Here the full scans also covered the 60 to 800 Da range with −10 and 10 eV collision energy and an acquisition time of 150 ms. Generally, the IDA data were used to identify the metabolites, but for those for which no MS^2^ spectra were included in the IDA acquisition, the SWATH data were used.

The UPLC-qToF-System was controlled by Analyst^®^ TF Software 1.7 (AB Sciex Pte. Ltd, Singapore) and the data were analyzed with Sciex OS 1.5.0.23389 (AB Sciex Pte. Ltd, Singapore/), MetabolitePilote^TM^ v. 2.0 (AB Sciex Pte. Ltd, Singapore), and MarkerView^TM^ v. 1.3.1 software (AB Sciex Pte. Ltd, Singapore).

### 3.4. Data Evaluation and Illustration

The data obtained from the IDA measurements were processed as follows.

To determine excretion kinetics of the detected metabolites, the peak areas obtained from the TOF-MS measurements were normalized with respect to the weights of the corresponding urine samples and plotted, in the form of area times dilution factor (y) values, against the time points when the samples were collected after coffee intake (x values, in hours).

The obtained kinetics of the single metabolites are semi-quantitative or relative evaluations because no standards were included in the measurements, which would have allowed determination of concentrations of the single metabolites.

Exact masses were assigned to the individual metabolites using the MS^2^ spectra obtained, in conjunction with published data and spectral predictions. The SWATH data were used if no MS^2^ spectrum of the metabolites was included in the IDA data. The recorded MS^2^ spectra and the assignment of the fragments are shown in the Supplementary Materials.

The metabolites were searched for using a library in SciexOS based on the structures and masses known in the literature and on the other hand masses were extracted, which showed a significant difference between the first two points in time. Furthermore, for 2-MF derived adducts analogous reactions as for furan were assumed and explicitly searched for this.

## 4. Conclusions

The presented results show that the UPLC-qToF-MS/MS approach can identify metabolites in a complex matrix such as urine rapidly and robustly without extensive sample preparation. Using it, we identified both metabolites that have already been identified in human urine samples and others that have only been previously detected in animal studies or theoretically postulated. Moreover, we obtained information on the excretion kinetics of x identified metabolites following the single intake of a coffee brew, and kinetics observed for y metabolites matched those obtained in previous targeted analyses.

Although these data provide a good overview of furan and 2-MF metabolites excreted in urine after the intake of a coffee brew, the study has several limitations. Quantification of the metabolites, and thus balancing of the total excretion of the furan and 2-methylfuran taken in from a coffee brew, is almost impossible with the available data, and at least one standard compound would have to be added to the samples for calibrated quantification. In addition, the subjects’ drinking behavior, and thus their urine volumes, varied substantially. Standardization of the urine’s dilution factors, e.g., using creatinine concentrations, would be helpful for enhancing the approach.

In summary, the new UPLC-qToF-MS/MS approach has several advantages that the targeted analysis of metabolites in a complex matrix such as urine lacks. If the analytical goal is considered when acquiring the samples, it can potentially provide abundant additional information in future animal or human intervention studies. The opportunities it provides for statistical evaluation of the data and rapid identification of metabolites responsible for differences in kinetic profiles of samples may be particularly valuable.

## Figures and Tables

**Table 1:** Exact mass, found mass, and retention time of each furan and 2-methylfuran metabolite identified by UPLC-qToF-MS/MS, and matrices in which they have been previously found (with supporting references). Meanings of the abbreviated metabolites’ names are given in the text or present the meanings in a footnote.

Metabolite	Chemical Formula	Exact Mass (Da)	Found at *m/z*	Retention Time (min)	Matrices where Previously Found and References
GSH-BDA	C_14_H_17_N_3_O_6_S	355.083809	356.1000 [M + H]^+^	7.63	human urine [19,20,21], urine, bile, and hepatocytes of rats [22]
Lys-BDA	C_10_H_16_N_2_O_3_	212.11609	213.1232 [M + H]^+^	2.27	human urine [20,23]
AcLys-BDA	C_12_H_18_N_2_O_4_	254.12666	255.1345 [M + H]^+^	4.36	human urine [20,23]; rat urine [19,23]
AcCys-BDA-Lys	C_15_H_23_N_3_O_5_S	357.13584	356.1167 [M − H]^−^	7.58	human urine and rat urine [23]; rat urine [23,24]; rat hepatocytes [22]
AcCys-BDA-AcLys	C_17_H_25_N_3_O_6_S	399.14641	400.1471 [M + H]^+^	1.24	urine, bile, and hepatocytes of rats [19,22,23,24]
AcCys (SO)-BDA-AcLys	C_17_H_25_N_3_O_7_S	415.14132	416.1484 [M + H]^+^	4.46	rat urine [19,23,24]
methanethiol-BDA-Glu	C_10_H_13_NO_4_S	243.05653	244.0793 [M + H]^+^	1.05	rat urine [19,24]
GSH-BDA-Lys	C_20_H_31_N_5_O_8_S	501.18933	502.1740 [M + H]^+^	7.57	rat urine [25]; rat hepatocytes [19,22]
GSH-BDA-Gln	C_19_H_27_N_5_O_9_S	501.15295	500.1478 [M −H]^−^	7.59	rat urine [25]; rat hepatocytes [19,22]
GSH-BDA-AcLys	C_22_H_33_N_5_O_9_S	543.19990	544.2036 [M + H]^+^	7.59	rat hepatocytes [19,22]
GSH-BDA-Glu	C_19_H_26_N_4_O_10_S	502.13696	503.1497 [M + H]^+^	1.47	rat bile [19,22,26]
Cys-BDA-GSH	C_17_H_24_N_4_O_8_S_2_	476.10356	475.0941 [M − H]^−^	7.68	rat bile [19,26]
CysGyl-BDA-GSH	C_19_H_27_N_5_O_9_S_2_	533.12502	532.1480 [M − H]^−^	7.60	rat bile [19,26]
BDA-Lys-BDA	C_14_H_18_N_2_O_4_	278.12666	277.1187 [M − H]^−^	7.57	not previously reported, but postulated
Cys-BDA	C_7_H_9_NO_3_S	187.03031	188.0384 [M + H]^+^	3.32	not yet reported in the literature, only theoretical

**Table 2:** Exact mass, found mass, and retention time of each furan and 2-methylfuran metabolite identified by UPLC-qToF-MS/MS, and matrices in which they have been previously found (with supporting references). Meanings of the abbreviated metabolites’ names are given in the text or present the meanings in a footnote.

Metabolite	Chemical Formula	Exact Massbreeak//(Da)	Found at *m/z*	Retention Time (min)	Previously Detected in (References)
AcCys-BDA	C_9_H_11_NO_5_S	245.25234	246.0431 [M + H]^+^	1.46	not previously reported, but postulated
Lys-AcA	C_11_H_20_N_2_O_3_	228.14739	227.1386 [M − H]^−^	3.87	not previously reported, but postulated
AcLys-AcA	C_13_H_20_N_2_O_4_	268.30890	267.1258 [M − H]^−^	7.65	not previously reported, but postulated
AcCys-AcA	C_10_H_13_NO_5_S	259.05144	258.0441 [M − H]^−^	7.58	not previously reported, but postulated

## Supplementary Materials

The following materials are available online: Figure S1: MS/MS spectrum of Lys-BDA; Figure S2: MS/MS spectrum of AcLys-BDA; Figure S3: MS/MS spectrum of GSH-BDA; Figure S4: MS/MS spectrum of AcCys(SO)-BDA-AcLys; Figure S5: MS/MS spectrum of AcCys-BDA-Lys; Figure S6: MS/MS spectrum of GSH-BDA-Lys; Figure S7: MS/MS spectrum of GSH-BDA-Gln; Figure S8: MS/MS spectrum of GSH-BDA-AcLys; Figure S9: MS/MS spectrum of CysGly-BDA-GSH; Figure S10: MS/MS spectrum of BDA-Lys-BDA; Figure S11: MS/MS spectrum of Lys-AcA; Figure S12: MS/MS spectrum of AcLys-AcA; Figure S13: MS/MS spectrum of AcCys-AcA; Figure S14: Urinary metabolites of 2-methylfuran.

## References

[1] IARC (1995). Dry Cleaning, Some Chlorinated Solvents and Other Industrial Chemicals.

[2] National Center for Biotechnology Information 2-Methylfuran. https://pubchem.ncbi.nlm.nih.gov/compound/2-methylfuran.

[3] European Food Safety Authority (2010). Update of results on the monitoring of furan levels in food. EFSA J..

[4] Altaki M., Santos F.J., Galceran M. (2011). Occurrence of furan in coffee from Spanish market: Contribution of brewing and roasting. Food Chem..

[5] Fromberg A., Fagt S., Granby K. (2009). Furan in heat processed food products including home cooked food products and ready-to-eat products. EFSA Support. Publ..

[6] Knutsen H.K., Alexander J., Barregård L., Bignami M., Brüschweiler B., Ceccatelli S., Cottrill B., Dinovi M., Edler L., Grasl-Kraupp B. (2017). Risks for public health related to the presence of furan and methylfurans in food. EFS2.

[7] Locas C.P., Yaylayan V.A. (2004). Origin and mechanistic pathways of formation of the parent furan—A food toxicant. J. Agric. Food Chem..

[8] Limacher A., Kerler J., Davidek T., Schmalzried F., Blank I. (2008). Formation of furan and methylfuran by maillard-type reactions in model systems and Food. J. Agric. Food Chem..

[9] Adams A., Bouckaert C., Van Lancker F., De Meulenaer B., De Kimpe N. (2011). Amino acid catalysis of 2-alkylfuran formation from lipid oxidation-derived α,β-unsaturated aldehydes. J. Agric. Food Chem..

[10] Neuwirth C., Mosesso P., Pepe G., Fiore M., Malfatti M., Turteltaub K., Dekant W., Mally A. (2012). Furan carcinogenicity: DNA binding and genotoxicity of furan in rats in vivo. Mol. Nutr. Food Res..

[11] National Toxicology Program (1993). Toxicology and Carcinogenesis Studies of Furan (CAS No. 110-00-9) in F344 rats and B6C3F1 mice (gavage studies). Natl. Toxicol. Program Tech. Rep. Ser..

[12] De Conti A., Kobets T., Escudero-Lourdes C., Montgomery B., Tryndyak V.P., Beland F.A., Doerge D.R., Pogribny I.P. (2014). Dose- and time-dependent epigenetic changes in the livers of fisher 344 rats exposed to furan. Toxicol. Sci..

[13] Peterson L.A., Naruko K.C., Predecki D.P. (2000). A Reactive metabolite of furan,cis-2-butene-1,4-dial, is mutagenic in the ames assay. Chem. Res. Toxicol..

[14] Palmen N., Evelo C.T. (1996). Glutathione depletion in human erythrocytes and rat liver: A study on the interplay between bioactivation and inactivation functions of liver and blood. Toxicol. Vitr..

[15] Ravindranath V., Burka L., Boyd M. (1984). Reactive metabolites from the bioactivation of toxic methylfurans. Science.

[16] Gill S.S., Kavanagh M., Cherry W., Barker M., Weld M., Cooke G.M. (2013). A 28-day Gavage Toxicity Study in Male Fischer 344 Rats with 2-methylfuran. Toxicol. Pathol..

[17] Kremer J.I., Gömpel K., Bakuradze T., Eisenbrand G., Richling E. (2018). Urinary excretion of niacin metabolites in humans after coffee consumption. Mol. Nutr. Food Res..

[18] Kremer J.I., Pickard S., Stadlmair L.F., Glaß-Theis A., Buckel L., Bakuradze T., Eisenbrand G., Richling E. (2019). Alkylpyrazines from coffee are extensively metabolized to pyrazine carboxylic acids in the human body. Mol. Nutr. Food Res..

[19] Moro S., Chipman J.K., Wegener J.W., Hamberger C., Dekant W., Mally A. (2012). Furan in heat-treated foods: Formation, exposure, toxicity, and aspects of risk assessment. Mol. Nutr. Food Res..

[20] Kremer J.I., Karlstetter D., Klier C., Rohtermund J., Thomas H., Becker H., Lang L., Bakuradze T., Eisenbrand G., Richling E. (2020). Detection and stability of BDA-lysine and -glutathione metabolites as urinary exposure biomarkers for furan in humans. Naunyn-Schmiedebergs Arch. Pharmacol..

[21] Peterson L.A., Cummings M.E., Chan J.Y., Vu C.C., Matter B.A. (2006). Identification of acis-2-butene-1,4-dial-derived glutathione conjugate in the urine of furan-treated rats. Chem. Res. Toxicol..

[22] Lu D., Sullivan M.M., Phillips M.B., Peterson L.A. (2009). Degraded protein adducts ofcis-2-butene-1,4-dial are urinary and hepatocyte metabolites of furan. Chem. Res. Toxicol..

[23] Grill A.E., Schmitt T., Gates L.A., Lu D., Bandyopadhyay D., Yuan J.M., Murphy S.E., Peterson L.A. (2015). Abundant rodent furan-derived urinary metabolites are associated with tobacco smoke exposure in humans. Chem. Res. Toxicol..

[24] Kellert M., Wagner S., Lutz U., Lutz W.K. (2008). Biomarkers of furan exposure by metabolic profiling of rat urine with liquid chromatography-tandem mass spectrometry and principal component analysis. Chem. Res. Toxicol..

[25] Rietjens I.M.C.M., Recker T., Günther H., Hanlon P., Honda H., Mally A., O’Hagan S., Scholz G., Seidel A., Swenberg J. (2018). Exposure assessment of process-related contaminants in food by biomarker monitoring. Arch. Toxicol..

[26] Hamberger C., Kellert M., Schauer U.M., Dekant W., Mally A. (2010). Hepatobiliary toxicity of furan: Identification of furan metabolites in bile of male F344/N rats. Drug Metab. Dispos..

[27] World Medical Association WMA Declaration of Helsinki: Ethical Principles for Medical Research Involving Human Subjects. https://www.wma.net/policies-post/wma-declaration-of-helsinki-ethical-principles-for-medical-research-involving-human-subjects/.

