# Supplementary material for: A New UPLC-qTOF Approach for Elucidating Furan and 2-Methylfuran Metabolites in Human Urine Samples after Coffee Consumption
Source: Molecules. 2020 Nov 3;25(21):5104. doi: 10.3390/molecules25215104 (PMC7663408; doi:10.3390/molecules25215104)
Supplement: Supplementary file 1 [file molecules-25-05104-s001.pdf]

Supplementary Material

In the supplementary material the obtained MS^2 spectra of the metabolites are shown.

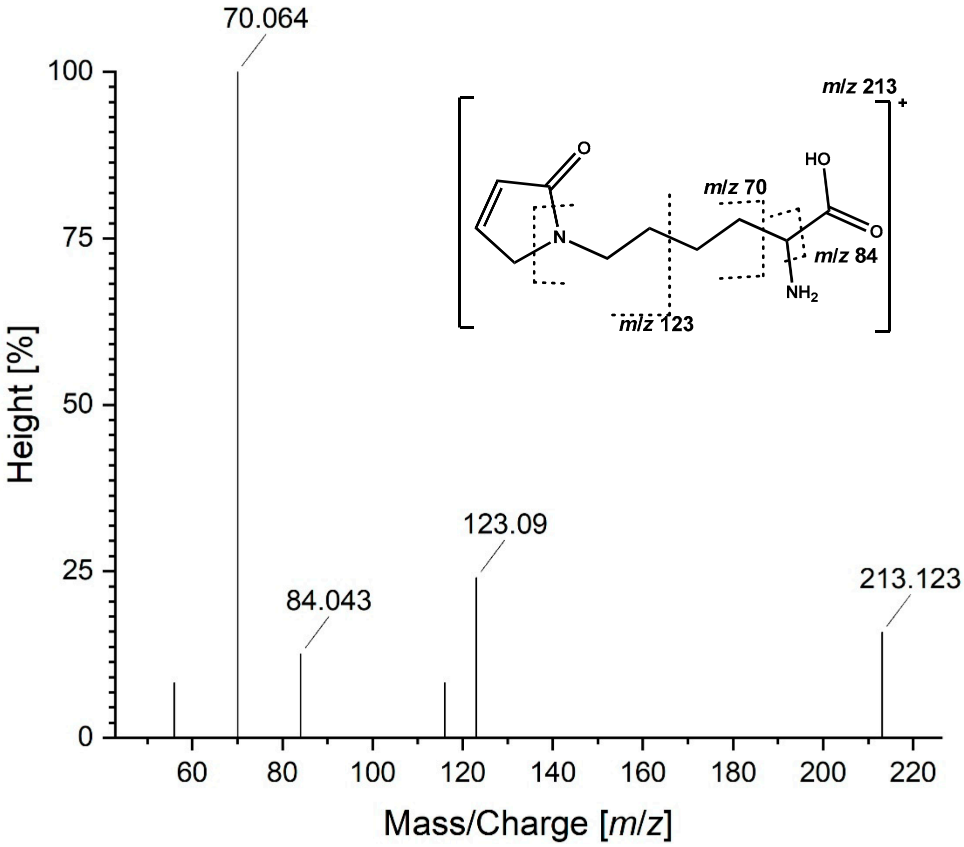

Figure S1: MS/MS-spectrum of Lys-BDA

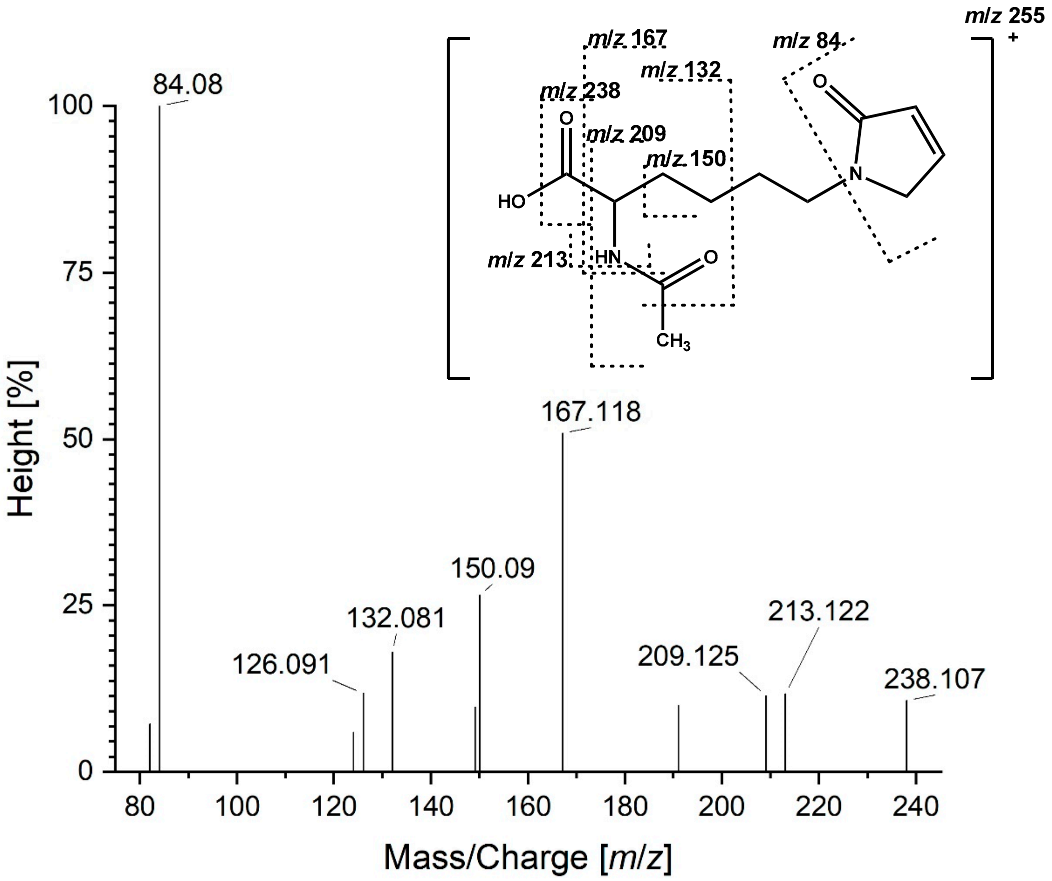

Figure S2: MS/MS-spectrum of AcLys-BDA

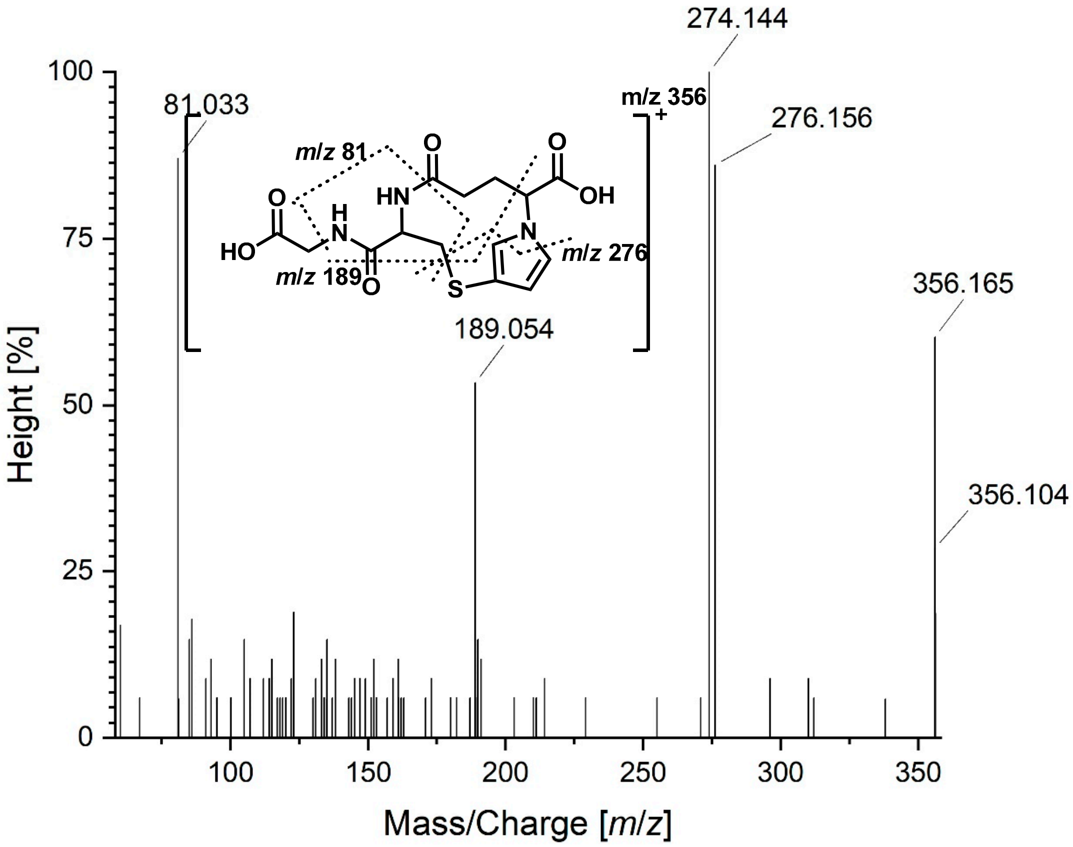

Figure S3: MS/MS-spectrum of GSH-BDA

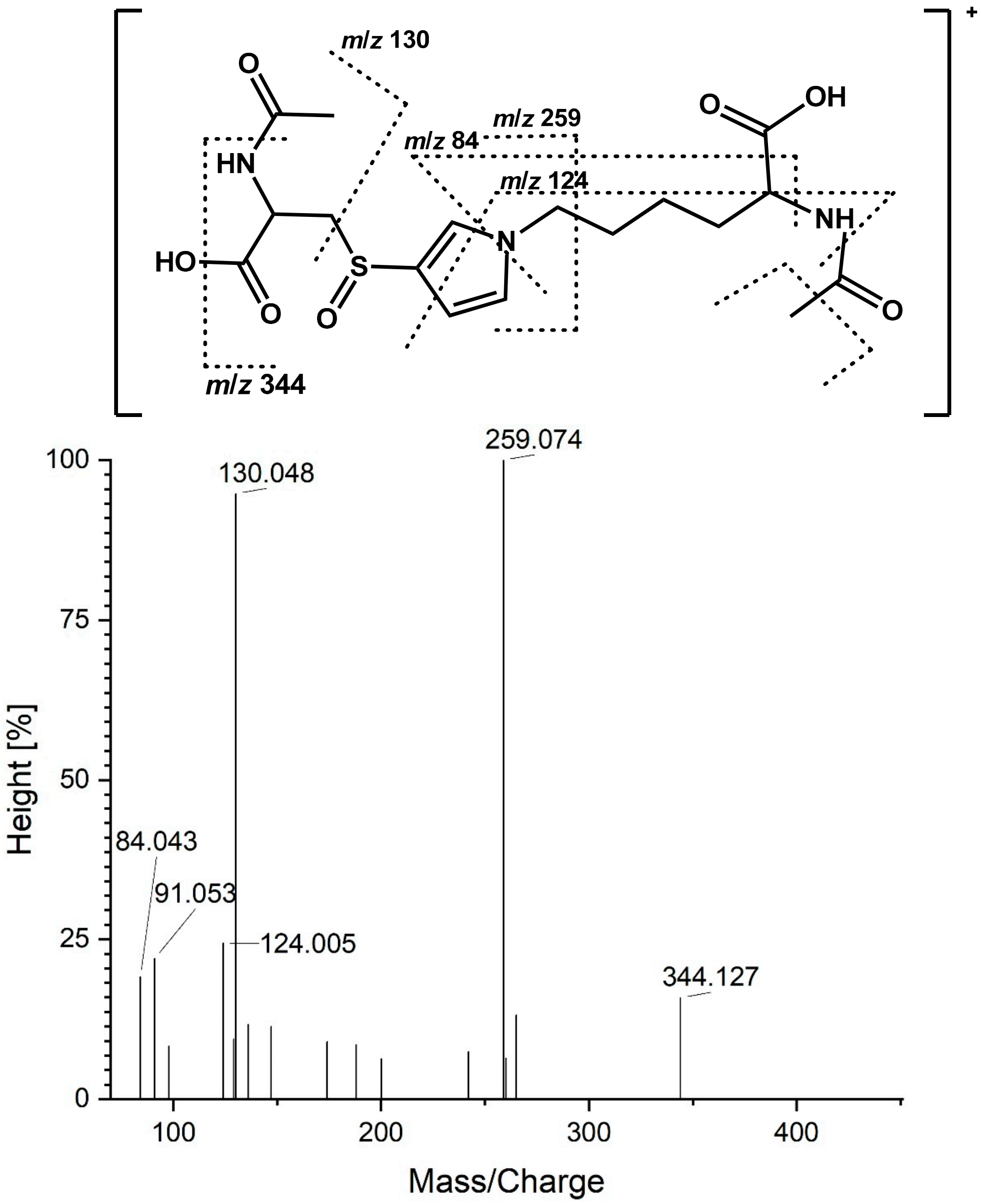

Figure S4: MS/MS-spectrum of AcCys(SO)-BDA-AcLys

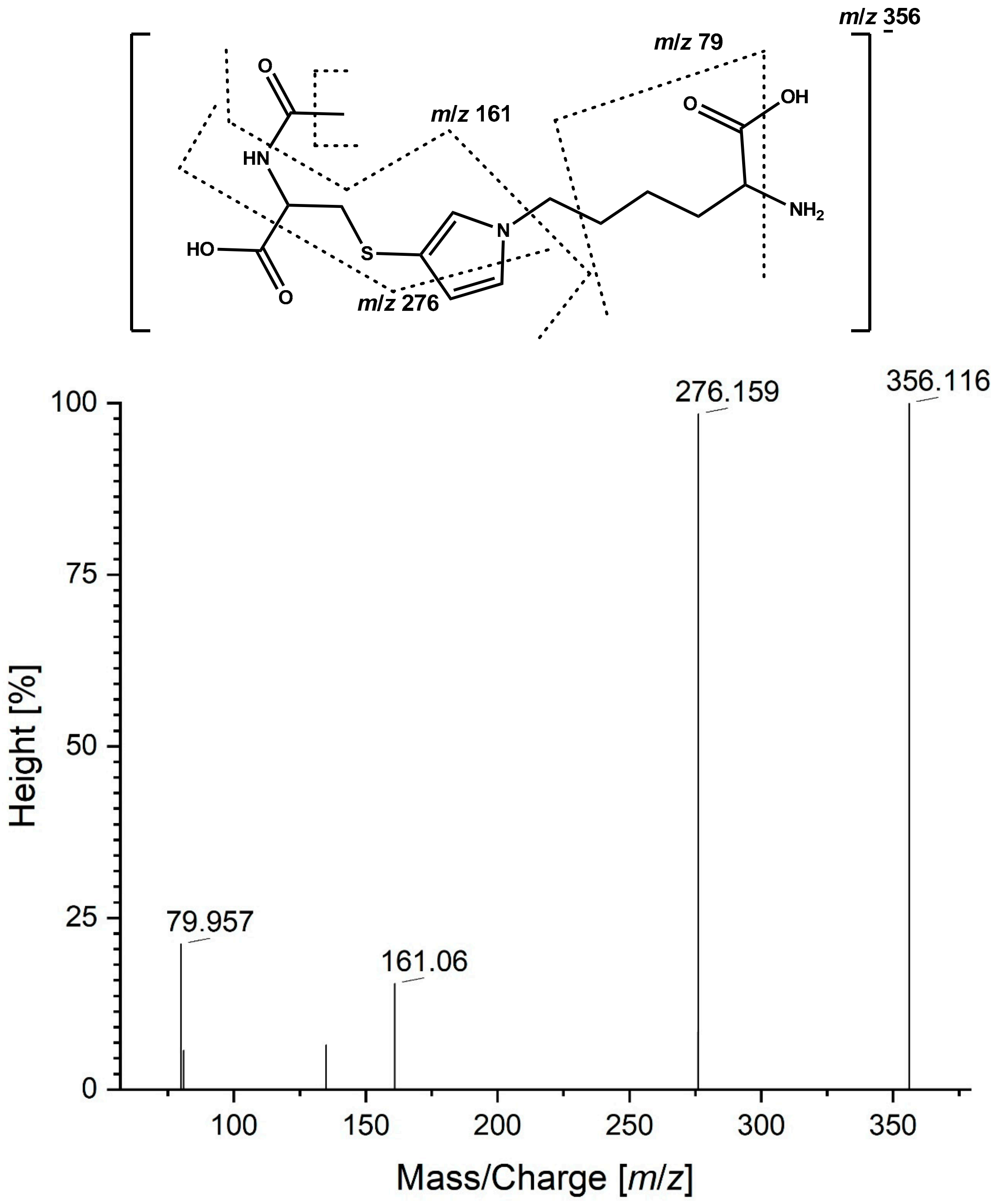

Figure S5: MS/MS-spectrum of AcCys-BDA-Lys

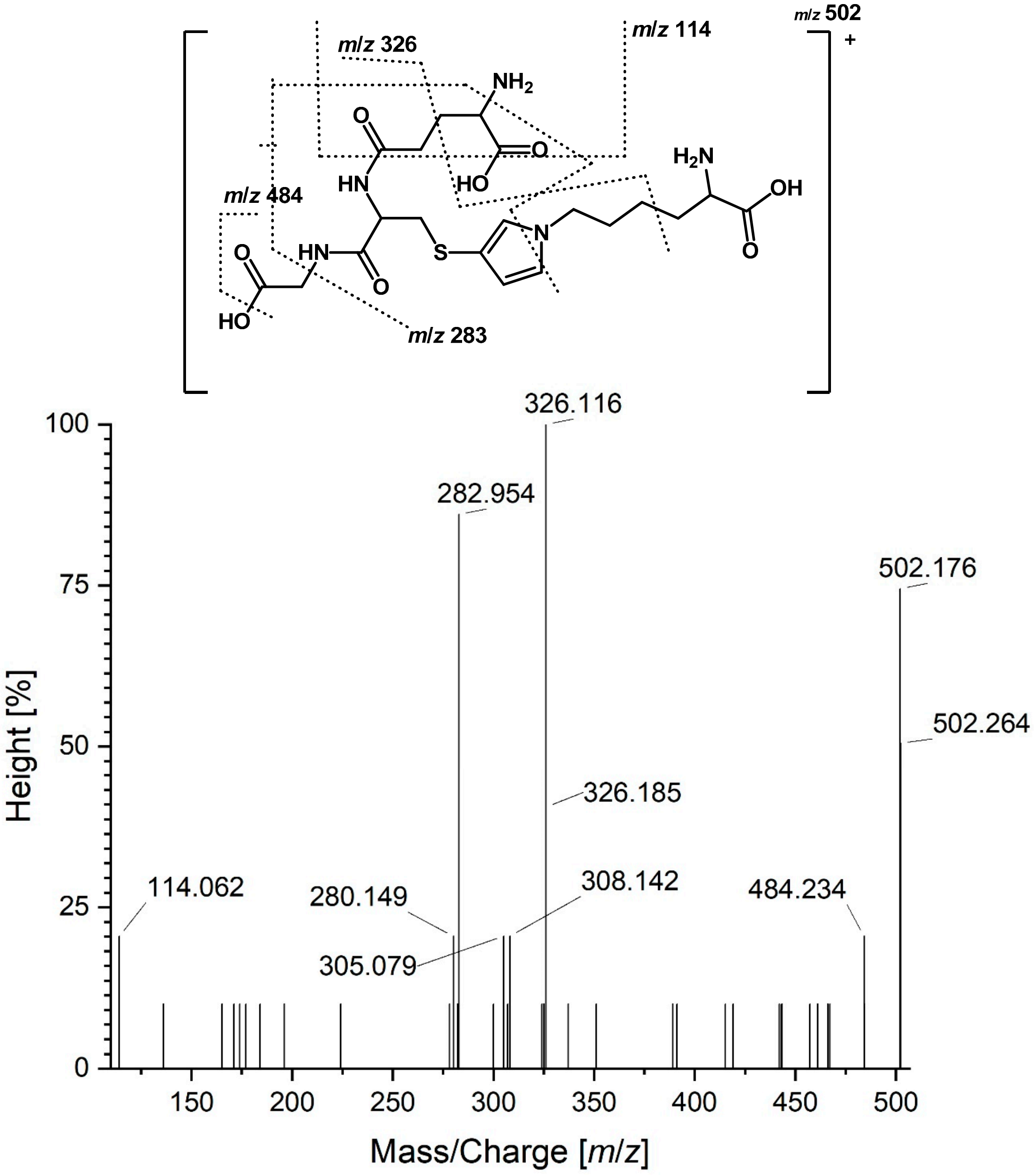

Figure S6: MS/MS-spectrum of GSH-BDA-Lys

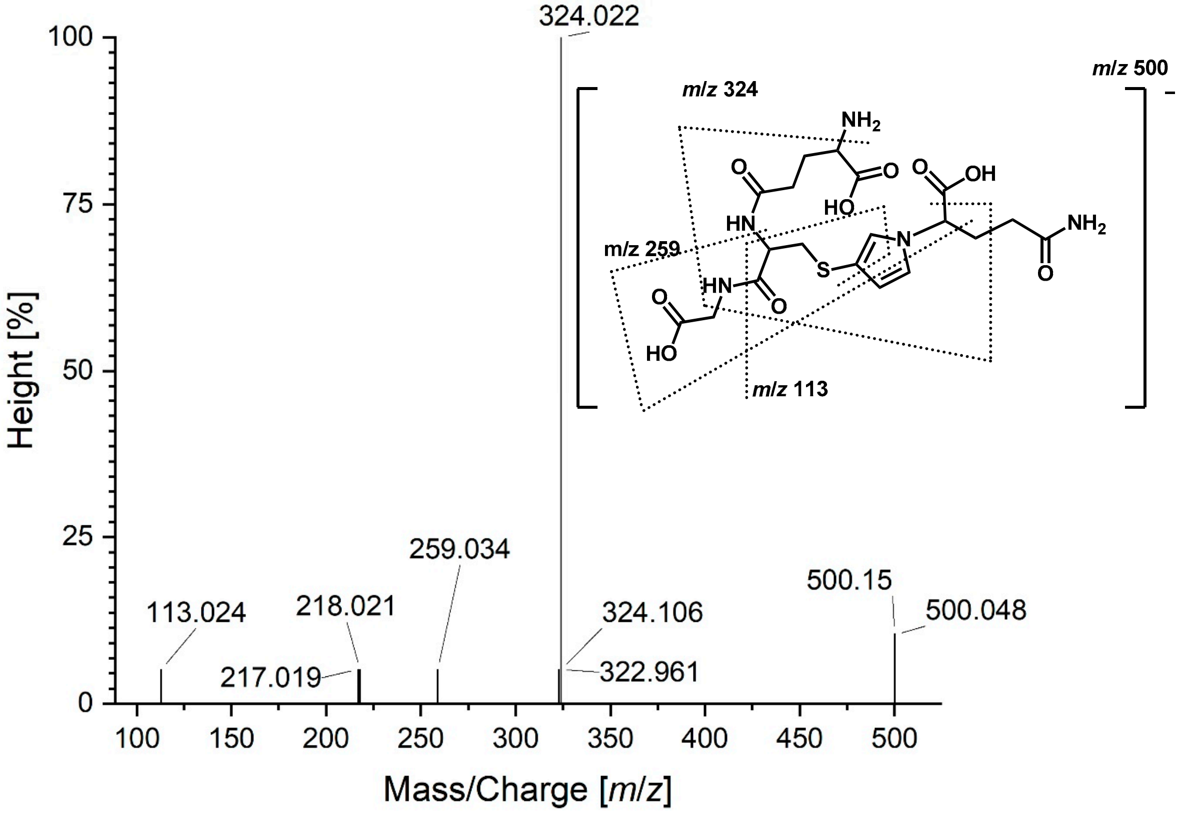

Figure S7: MS/MS-spectrum of GSH-BDA-Gln

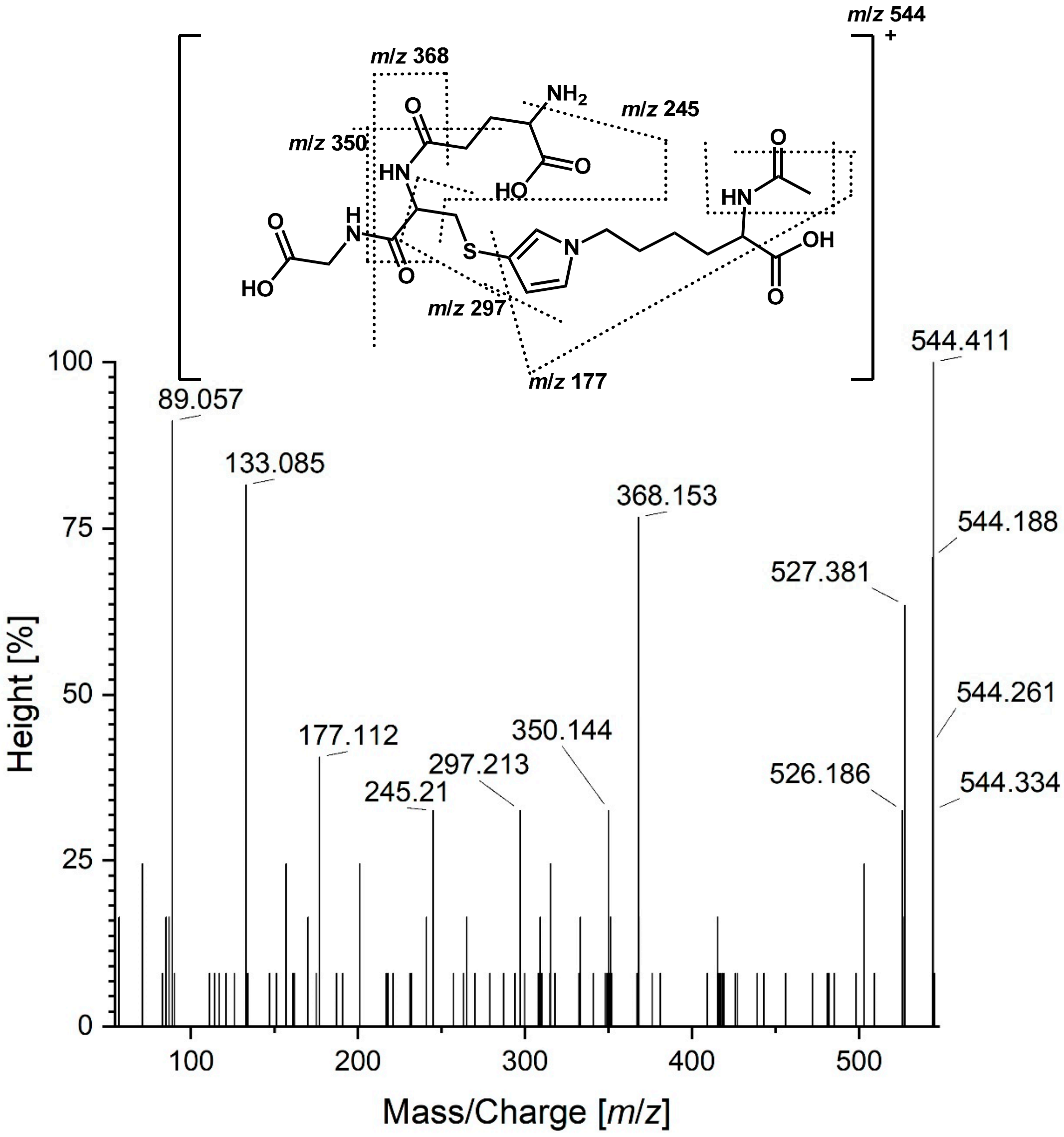

Figure S8: MS/MS-spectrum of GSH-BDA-AcLys

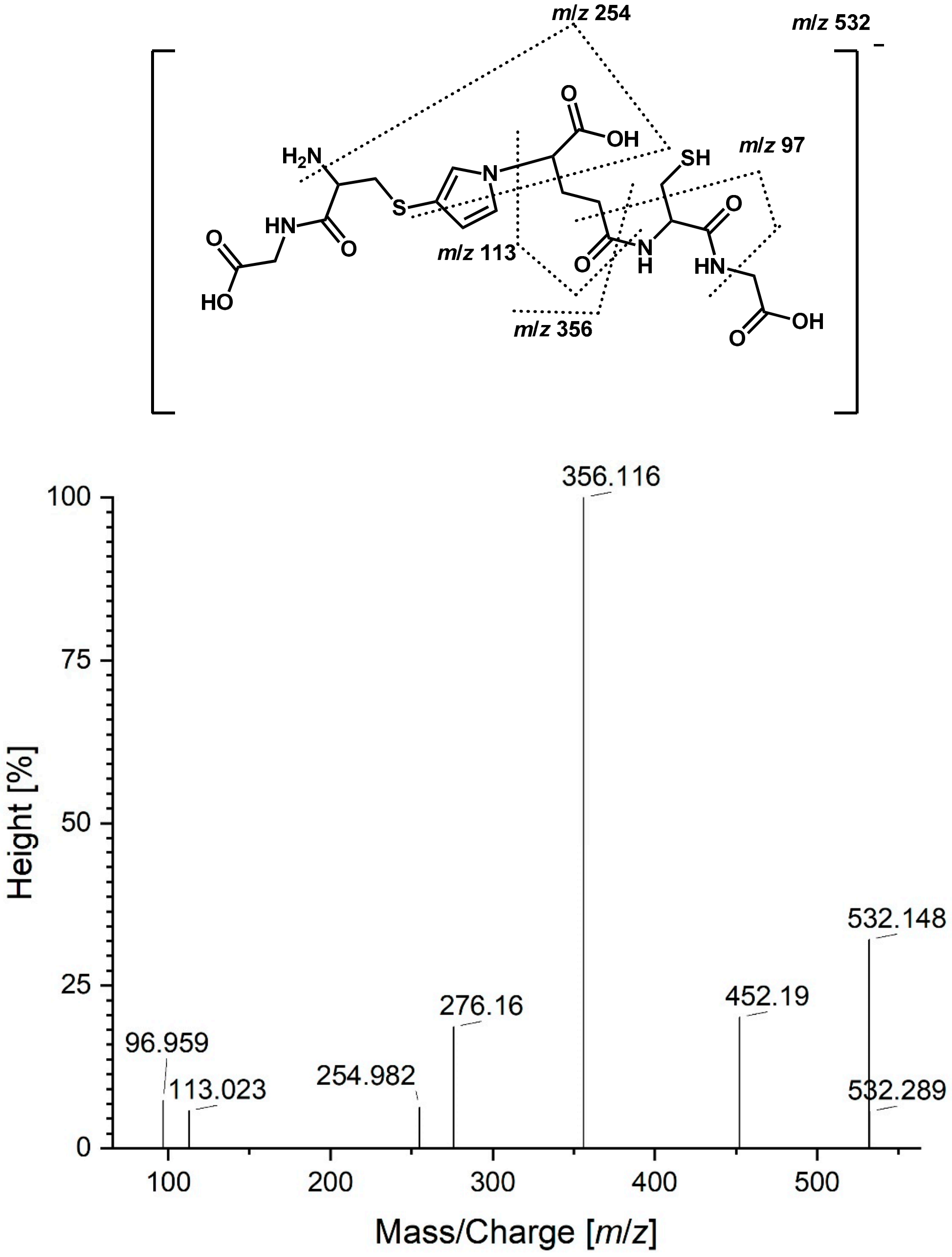

Figure S9: MS/MS-spectrum of CysGly-BDA-GSH

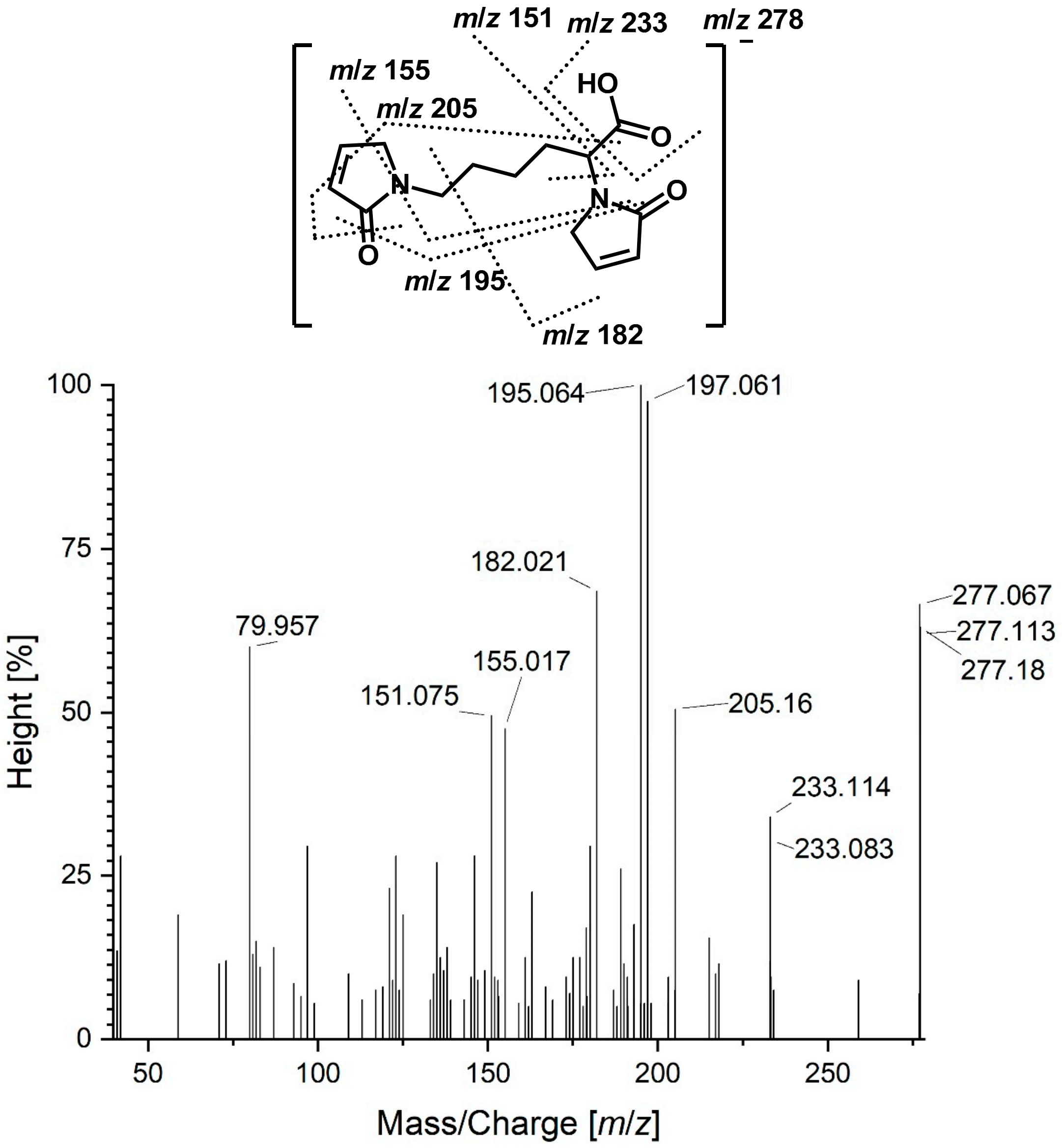

Figure S10: MS/MS-spectrum of BDA-Lys-BDA

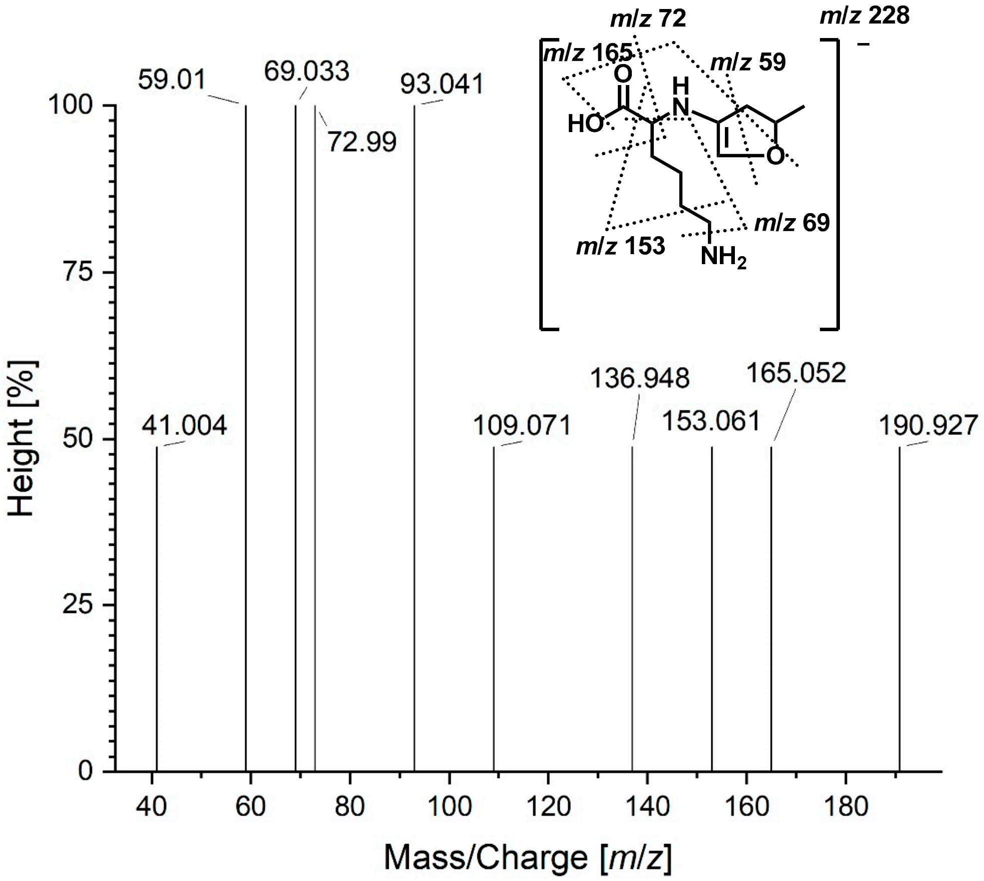

Figure S11: MS/MS-spectrum of Lys-AcA

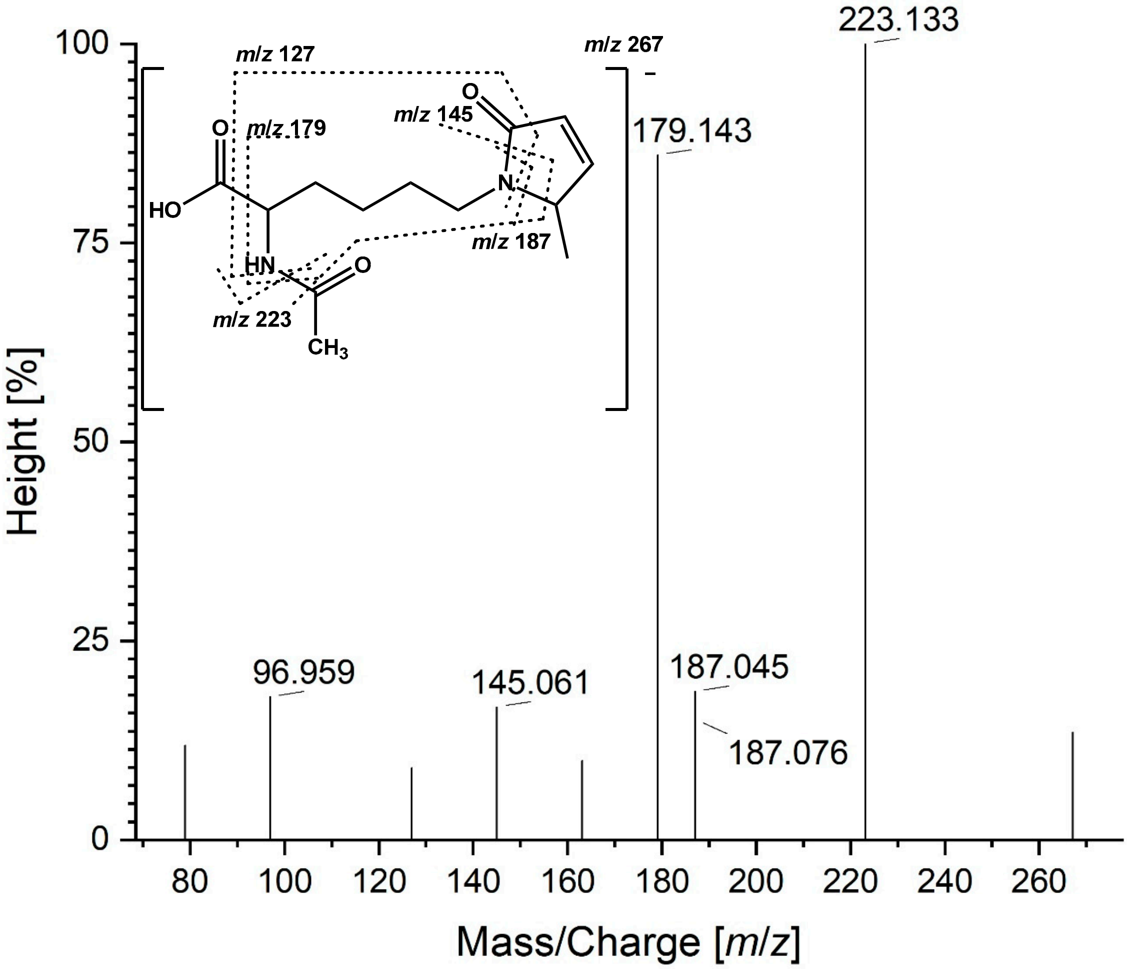

Figure S12: MS/MS-spectrum of AcLys-AcA

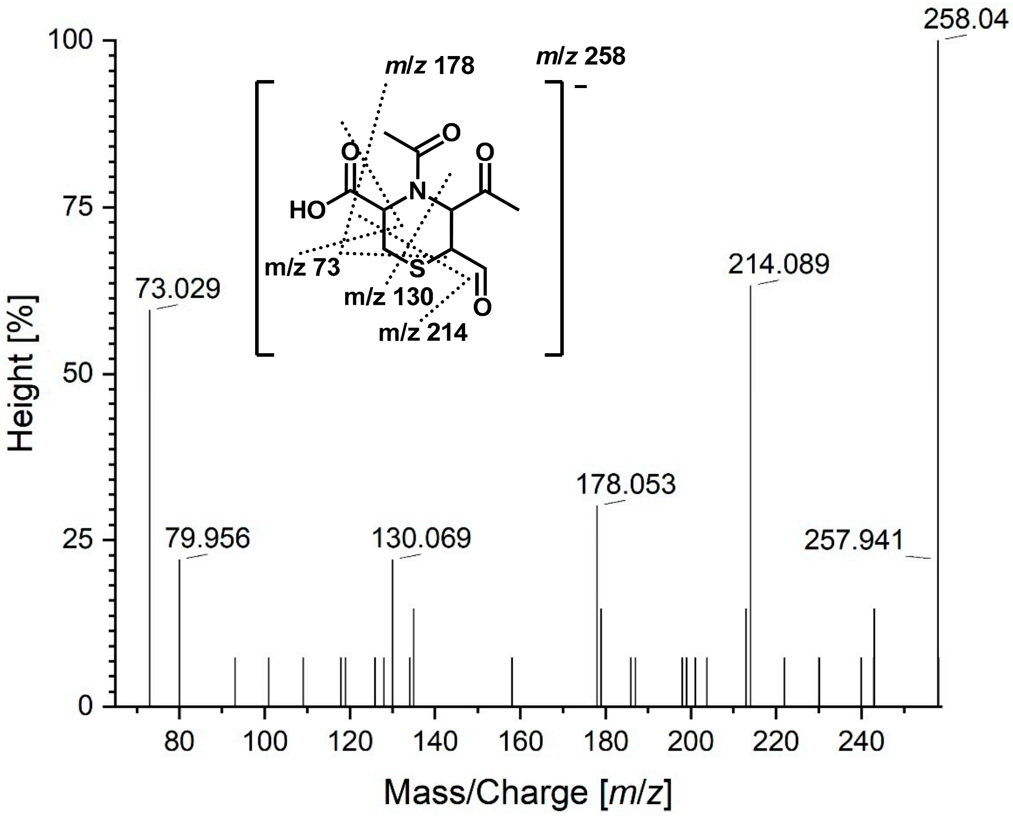

Figure S13: MS/MS-spectrum of AcCys-AcA

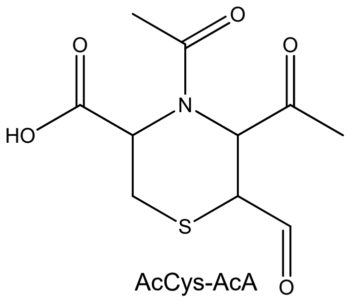

AcCys-AcA

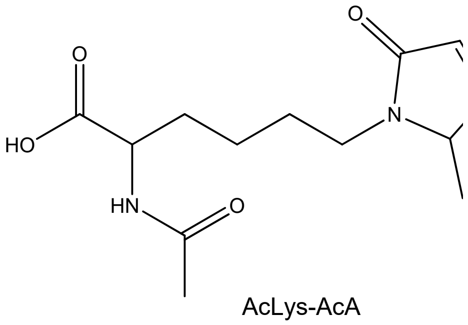

AcLys-AcA

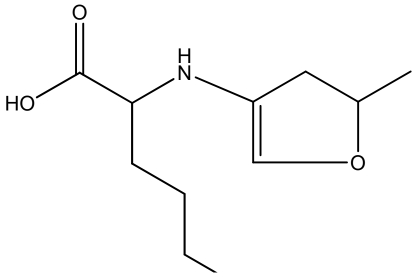

Figure S14: Urinary metabolites of 2-methylfuran.
